# Prevalence of Supernumerary Teeth in Patients with Cleft lip and Palate through Orthopantomographic Analysis: A Systematic Review: Prevalence of Supernumerary Teeth in Patients with Cleft lip and Palate

**DOI:** 10.31661/gmj.v13iSP1.3654

**Published:** 2024-12-08

**Authors:** Abbas Salehi Vaziri, Atefe Ahmadvand, Anahita Dehghani Soltani, Seyed Masoud Sajedi, Hossein Shahoon

**Affiliations:** ^1^ Department of Orthodontics, School of Dentistry, Shahed University, Tehran, Iran; ^2^ Department of Orthodontics, Faculty of Dentistry, Shahed University, Tehran, Iran; ^3^ Department of Orthodontics, School of Dentistry, Shahid Beheshti University of Medical Sciences, Tehran, Iran; ^4^ Department of Oral and Maxillofacial Medicine, Faculty of Dentistry, Shahed University, Tehran, Iran; ^5^ Oral and Maxillofacial Surgery Department, Faculty of Dentistry, Shahed University, Tehran, Iran

**Keywords:** Supernumerary Teeth, Cleft Lip, Palate, Orthopantomo Graphic, Hospital Infection

## Abstract

**Background:**

One of the most common injuries in the jaw and face area is cleft lip and palate, which causes an increase in the size, shape and time of formation and growth of teeth. Early detection of the number of missing teeth and paying attention to the size, shape and number of the remaining teeth is one of the goals of this study.

**Materials and Methods:**

The present study investigated the issue by reviewing 45 articles with keywords including “Supernumerary teeth”, “Cleft lip”, “Palate”, “Orthopantomo graphic” and “Hospital Infection” in 2012 to 2024.

**Results:**

The most common abnormality was hypoxemia, followed by malocclusion and hyperemia. The most cases of hypoxemia were observed in bilateral cleft lip and palate, and the lowest cases were found in single cleft lip. Many problems that arise in the mouth and teeth can be solved by observing health and care tips and also by teaching children how to brush their teeth correctly, but one of the things that is necessary and necessary for children’s health is regular and periodic dental visits. In these visits, many oral and dental problems are revealed and they can be solved before more problems occur and high dental costs can be avoided. One of the problems of extra teeth in children is that if it is identified in the early stages, it can be treated and damage to other teeth can be prevented.

**Conclusion:**

Extra teeth may cause delayed growth or non-growth of adjacent teeth. In addition, excessive retention of baby teeth, deformation of the roots of adjacent permanent teeth, displacement of teeth, creation of an unnatural distance between teeth, root erosion of adjacent teeth, or the formation of cysts around extra teeth can be among the complications that occur in the presence of teeth. They encountered extra.

## Introduction

Unlike other types of submucosal cleft palate, it is not diagnosed at birth [1]. In this case, there is a gap in the roof of the mouth (hard palate or soft palate), but it is not obvious. In this case, the skin covers the roof of the mouth over the gap [2]. People with hidden cleft or submucosal cleft palate do not have enough ability to prevent air from escaping from the mouth to the nose when speaking. Inadequacy of their palatopharyngeal valve raises the suspicion of submucosal cleft palate [3]. In some cases, experts can find their submucous gap by examining the roof of the mouth through a finger or other cases. The biggest problem of these people is the quality of nasal speech [4].

### Symptoms of Cleft Palate and Lip

In most cases, the presence of cracks along the lip is the most obvious and main symptom of diabetes. In this situation, milk may come out of your baby’s nose while feeding. Because the barrier between mouth and nose in these children is abnormal. In children with this birth defect, there may be problems related to teeth. For example, they may have loose teeth or their number of teeth is more or less. Also, irregular teeth are very common in these people. Cleft palate can also cause frequent middle ear infections and Eustachian tube problems in your child. The Eustachian tube is a duct that helps drain fluids from the ear and adjusts the pressure on both sides of the eardrum. If your child has a problem with an ear infection and the Eustachian tubes are not draining properly, the child may have hearing loss. If your child has cleft lip or cleft palate, he may have difficulty speaking and pronouncing different words. Speech problems caused by clefts usually involve nasal or intranasal sounds [5].

### Types of Extra Teeth

Extra teeth can come in many forms, but four types are more common than others. In the following, we explain these four types:

1- Mesiodens teeth: Mesiodens teeth, which are known as the most common type of extra teeth in the upper jaw, usually grow behind the front teeth. These teeth may, due to the incorrect position of other teeth, require the extraction of additional teeth or the use of mobile orthodontics or fixed orthodontics to adjust the front teeth [6].

2- Dissimilar and paramolar teeth: Distomolars and paramolars refer to extra molars that may appear in the area of ​​third molars (wisdom teeth). These extra teeth may lead to problems such as jaw tightness and crooked teeth and require surgery or orthodontic treatment [7].

3- Natal teeth: Natal teeth are the teeth that babies are born with. These extra milk teeth are usually seen in the area of ​​the milk teeth and may need special care and examination. Treatment for any extra teeth may include extractions [8], orthodontics, or special care to prevent future problems. Accurate diagnosis and consultation with a specialist dentist is necessary to choose the right treatment method [5].

### Causes of Extra Teeth

Several reasons can lead to the appearance of extra teeth in childhood and adulthood, including:

1- Fabry disease: People with Fabry disease cannot produce certain enzymes to break down fatty substances. One of the complications of this disorder is the appearance of extra teeth in the mouth.

2- Cleft lip and palate: A cleft lip or palate, which is a congenital anomaly, can affect the growth of teeth and cause extra teeth to appear in the upper or lower jaw. This problem occurs during pregnancy and due to defects in the development of the baby’s palate [9].

3- Gardner’s syndrome: Gardner’s syndrome is a rare hereditary disorder that leads to the abnormal growth of tissues and the creation of extra teeth. This syndrome can also cause benign tumors.

4- Cleidocranial dysplasia: this rare genetic disease affects the growth of bones and teeth and can cause deformed bones, malformed teeth and extra teeth [10].

5- Ehlers-Danlos syndrome: Ehlers-Danlos syndrome, which affects the connection of connective tissues, may lead to the growth of extra teeth in the gums.

### Causes of Cleft Palate and Lip

The cause of cleft lip and palate is not yet known precisely, but doctors believe that this defect occurs due to the effects of genetic and environmental factors. If one or both parents carry the defective gene related to the development of this defect, it is possible that their child will also have this defect. In addition to genetic factors, researchers believe that doing some things during pregnancy can have an effect on the development of cleft lip and palate in the fetus [6].

### Problems of Children with Cleft Palate and Lip

Alveolar bone graft (jaw): Children who have cleft lip and palate, which also includes the alveolar dental arch gap, should perform a bone graft, so that their dental arch is preserved and the teeth can grow in this area. The timing of this surgery varies, but it is usually performed around six to eight years of age. First, an X-ray photo is taken of the mouth, to determine the growth status of the permanent teeth. After the dental team has aligned the tooth tissue, or the cleft lip has been closed, the cancellous bone of the estigh Khasre is placed in the jawbone. Usually, the child will stay overnight in the hospital to make sure he has received enough fluids and is in a stable condition. Discomfort from hip pain and unwillingness to walk are common in these conditions. A soft diet and restriction of intense activities are recommended for up to 10 days.

Feeding problems in children with cleft lip and palate: Many children who have this problem cannot breastfeed easily. Because the gap in the roof of their mouth makes them unable to create enough suction in their mouth. Therefore, a large amount of air and milk enters their nose. These children have difficulty gaining weight in the first few months of life [7]. A nutritionist can teach parents alternative ways to feed a child. If breastfeeding is not possible, it may be recommended to pour breast milk into a flexible bottle designed for cleft palate children and give it to the baby. In rare cases, it may be necessary for the child to be fed through a tube placed inside the nose until the reconstructive surgery is performed.

Hearing problems of children with cleft lip and palate: In these children, there is a high probability of fluid accumulation inside the ear [8]. The reason is that the muscles of the roof of the mouth are connected to the middle ear, and if they do not work properly due to the cleft palate, sticky secretions of the ear may collect inside it and cause hearing problems. Audiometry should be done regularly to check any hearing problems. Hearing problems may improve after cleft palate repair. If necessary, it is possible to help drain the fluids by placing a small plastic tube inside the eardrum and solve this problem. Sometimes hearing aids may be recommended.

## Materials and Methods

**Figure-1:**
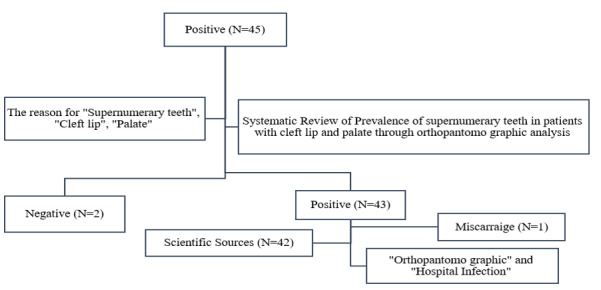
Figure 1. Flow chart of included subjects

Despite a number of demographic variables that could influence the direction or strength of this link, including "Supernumerary teeth", "Cleft lip", "Palate", "Orthopantomo graphic" and "Hospital Infection" in 2012 to 2024 systematic review highlighted a bidirectional association between depression and obesity.

### Dental Care in Cleft Lip and Palate Patients

The amount of dental problems in people with cleft lip and palate is more and more common than normal people. Children with cleft lip and palate should be under the supervision of a dentist, so that any dental problem caused by the cleft can be treated with the help of a dentist. Sometimes, due to the major problems of cleft lip and palate in these children [9], dental problems are not given much attention. Routine dental care is important for all children, but how the child’s teeth are positioned or the absence of teeth has an important effect on the success of treatment for cleft lip and palate patients. Some dental abnormalities of children with cleft palate and lip are:

* Absence of teeth.

* Rotation of the teeth.

* Misplaced teeth in the wrong place.

* Extra teeth.

* Teeth deviated towards the lip.

* Teeth deviated towards the tongue [10].

Children with cleft lip and palate may have specific dental problems related to the cleft. The gap may involve the gums and cause some teeth to be misplaced, misshapen, or not erupt at all. There may even be extra teeth in and around the gap. Usually, front teeth and upper canines that are around the gap are more affected. Dental problems of children with cleft palate should be followed up by experts in different fields of dentistry from birth. Dental specialists, including pediatric dentists, orthodontists, maxillofacial surgeons, and prosthetists help treat these patients. Many dentists recommend that the first dental examination be done at the age of 1, and even earlier if there are specific dental problems [11]. Routine care is performed by a dentist from the age of 3. Since children with cleft lip and palate are at a high risk of contracting dental diseases. Therefore, in such a situation, providing the necessary training and guidance as well as giving motivation and encouragement to the patient and his family is essential. Because this defect can lead to problems for milk and permanent teeth. Full compliance with the principles of oral and dental hygiene also helps to ensure healthy teeth in the future. So help your child start brushing at an early age and make sure his diet is full of nutritious foods. Your child’s dentist can advise you on the best ways to care for your teeth at a young age, including brushing techniques, fluoride use, and proper nutrition. Cleft palate is a common yet treatable birth defect. With a combination of several surgeries and corrective devices, the health and beauty problems caused by these gaps can be fixed or controlled.

## Findings

### Dental Treatment of Cleft Lip and Palate (Orthodontics)

Before the age of 12: According to the recommendations of experts, the first dental visit in these children should start at around one year of age or even earlier, and regular visits should start at around 3 years of age [12]. Orthodontic treatment is long-term and may continue up to the age of 21 in separate stages. The sooner this treatment is done, the more beneficial and less expensive it is. At birth, special orthodontic devices are made to fit the child’s mouth and placed in the gap, to prevent milk from coming back from the nose and to feed the child better. After the eruption of permanent teeth, orthodontics helps to regularize the teeth. The first step is to use removable orthodontics [13]. This stage usually starts at the age of 5 or 6 and continues until the age of 12. The goal of the first stage of orthodontics is to develop the dental arch in the upper jaw and align the front teeth and correct the abnormalities in the jaw. This action should be done before bone grafting, to create a good ground for teeth to grow in the gap. Treatment with movable orthodontics not only aligns the lower teeth, but also guides the upper jaw forward, to compensate for its growth retardation.

### Surgical Treatments for Cleft Palate and Lip

Cleft lip and palate surgery is performed in a hospital under general anesthesia. Usually, the sequence of surgeries is as follows:

1- Initial repair of cleft lip (3 to 6 months): In this operation, the surgeon creates tissue flaps by making cuts on both sides of the cleft. Then the flaps including the lip muscles are sewn together. This restoration makes the appearance, structure and function of the lips more natural. If needed, primary nose repair is also performed at the same time [14].

2- Cleft palate repair (up to 12 months of age or earlier if possible): Depending on the conditions, different methods are used to close the cleft palate and reconstruct the roof of the mouth. The surgeon makes incisions on both sides of the gap and moves and rearranges the tissues and muscles and closes the repair site with stitches [15].

### Follow-up Surgeries (between 2 Years and Late Adolescence)

1- Bone grafting: Bone grafting is usually recommended between the ages of 8 and 11 years. Bone grafting in the upper jaw is a standard treatment for cleft lip and palate. In this procedure, a small piece of bone is removed from another part of the body and placed in the gap. This operation is performed by a maxillofacial surgeon in order to provide nose and lip support, correct facial symmetry, create a natural tooth row in the upper jaw, improve the stability of the upper teeth and the roof of the mouth, and create a more natural appearance [16].

2- Dental implant: in cleft palate and lip deformity, some teeth are missing and the empty space of these teeth can be filled with a dental implant. A dental implant is a small base made of titanium that is implanted in the bone instead of the tooth root and is as strong as a natural tooth [17].

3- Placing the teeth in cleft palate and lip: It is better to remind that children with cleft palate are more at risk of tooth decay than others and they need to keep their teeth clean and, if necessary, fluoride treatment. After teeth sprouting, they should be cleaned with a toothbrush, and if this is not possible, washing can be done with the help of a sponge dipped in mouthwash solution [18].

4- Dealing with cleft lip and palate: Of course, no parent expects to give birth to a child with cleft palate and lip, and facing this situation can be emotionally exhausting for the whole family. If faced with this problem, parents should focus on supporting the child and helping him without blaming himself. The first step is to accept the problem and make the child understand that his personality has nothing to do with the existing problem. This work is facilitated with the help of a social worker and a psychologist [19].

### What is Craniofacial Orthodontics?

Craniofacial orthodontics is a trend of orthodontics that treats patients with congenital defects such as cleft lip and palate. An orthodontist, in cooperation with a speech pathologist, a pediatric dentist, an otolaryngologist, an oral surgeon, and a craniofacial plastic surgeon, prepares a suitable treatment plan for the correction of cleft lip and palate and the treatment of other jaw and facial abnormalities. A craniofacial orthodontic specialist evaluates the formation and growth of the teeth and jaw and performs non-surgical treatments to change the position of the jaws. This group of specialists also takes responsibility for the treatments before and after jaw surgery and monitors the growth process using radiography and molding [18].

### Additional Dental Treatment

Because extra teeth in the upper or lower jaw can negatively affect the beauty and health of the mouth and teeth, it is recommended to quickly consult an orthodontist and start treatment:

1- Extraction of extra teeth: If milk teeth are still left in the mouth at an older age, extracting these teeth and using orthodontics to adjust the jaw can be a suitable option for treatment [19].

### What are the Physical and Oral Health Challenges Related to Cleft Palate and Lip?

Some of the key physical and oral health challenges associated with cleft lip and palate include:

1- Facial abnormalities: One of the most obvious physical features is cleft palate [20], cleft in the upper lip and roof of the mouth. In some cases, the fissure extends into the nostril, resulting in a visible cleft in the nose [21].

2- Speech and swallowing problems: Clefts in the roof of the mouth can make it difficult for babies to breastfeed and for older children to speak clearly [22]. It can also affect the ability to swallow food properly and potentially lead to aspiration pneumonia [23].

### What Are the Symptoms of Hypertonia?

A clear sign of hypertonia is the appearance of one or more extra teeth, and in most cases hypertonia does not cause discomfort [24].

Causes of extra teeth: Experts are not sure exactly what causes hypertonia, but they have identified several factors that can contribute to the development of this disease, which include:

* Genetics and overactive dental lamina (cells responsible for tooth growth).

* Atavism, when an ancestral genetic trait reappears [25].

Causes and factors of the growth of extra teeth: Extra teeth can exist in various forms such as wedge-shaped, cone-shaped, button-shaped and complementary. Also, these teeth can grow in the front or back of the tooth or grow right next to the natural teeth. This situation sometimes causes a person to feel pain and has a destructive effect on his eating and speaking. No adverse effects have been reported in some children. There are several theories for the growth of extra teeth, which include:

* Heredity and genetics [26].

* Splitting the bud of one tooth into two halves and turning into two teeth.

* Excessive activity of the dental membrane and the tissue forming the tooth bud in the upper jaw.

* Some diseases such as cleft lip and palate, Gardner’s syndrome.

Extra tooth extraction in orthodontics: Extra teeth cause many problems for a person, the treatment of which is usually in the form of tooth extraction. If the extra tooth prevents the side teeth from coming out or causes crowding and cluttering of the teeth, then it should be treated immediately. The insertion of wisdom teeth, which is sometimes considered as one of these extra teeth, disrupts the order and good functioning of the teeth, and it is necessary to pull them out as soon as possible [27]. If an action is not taken for the extra tooth, in the future it is necessary to use orthodontics to correct the condition of the teeth. Sometimes, it is necessary to pull one or all extra teeth in order to open the space for the correct and proper movement of the teeth in order to perform orthodontics, and this tooth extraction cost is added to the total orthodontic cost.

Complications of extra teeth in oral health: An extra tooth causes problems for the teeth, which we will introduce some of them below:

* Occurrence of compression and misalignment of teeth.

* The possibility of a tumor or cyst [28].

* Prevents the natural growth of permanent teeth.

* The possibility of welding to the tooth and exposing it to wear.

* Adverse effect on the appearance of teeth.

* Damage to the roots of adjacent permanent teeth and their decay.

* They cause displacement of permanent teeth.

* They cause eating and speech disorders.

* It leads to infection, abscess and cavities.

### Causes of Extra Teeth in Children

1- Gardner’s syndrome: Gardner’s syndrome is another common problem in children, which is associated with the formation of large and numerous polyps in the intestine, benign tumors in the jaw and skin cysts [29]. This syndrome leads to the problem of extra teeth. Usually, there are no signs that indicate the presence of extra teeth in children’s mouths, and finding and diagnosing extra teeth usually happens by accident. When you take your child to the dentist for one of the following reasons:

* Displacement of permanent teeth: You may notice that your child’s permanent teeth are moving out of their original path. This displacement can involve moderate or complete rotation of the teeth [30].

* Speech problems: the presence of a large number of teeth in the mouth of a child with extra teeth affects the child’s ability to speak clearly [31].

2- Abnormal appearance of the face: baby teeth also have an effect on the appearance of your child’s face. The presence of a large number of teeth in the child’s mouth makes his face look abnormal.

### Treatment of Extra Teeth in Children

Some of the methods of diagnosis and treatment of extra teeth in children are as follows:

1- Regular dental check-ups: You should start your child’s dental check-ups before reaching the age of one year. In this way, the pediatric dentist can control your child’s oral health and treat extra teeth in the early stages of their development. If you leave the extra tooth alone, they can cause more damage to other teeth, whether milk or permanent [32].

2- Tooth extraction: Tooth extraction is the most effective way to treat extra teeth. Dentists only remove extra milk teeth if the teeth are loose and there is a possibility of choking. Because the tooth can enter the lung.

### What Are the Symptoms of Extra Teeth?

 Among the symptoms of extra teeth in the mouth are:

1- Abnormal tooth shape: One of the visible signs of this problem is the abnormal shape of permanent teeth [33].

2- Distance between the front teeth: The presence of a gap between the front teeth cannot always be a sign of a problem, but sometimes it is a sign of the presence of an extra tooth in the upper or lower gums.

3- Extending the growth time of the permanent tooth: If more than 6 months have passed since your child’s milk tooth fell out, but his permanent tooth has not yet grown. This problem can be due to the presence of extra teeth in the mouth, which prevents the growth of permanent teeth [34].

### Can an Extra Tooth be Used Instead of the Original Tooth?

The answer to this question depends on where the extra tooth is located. In some cases, the extra tooth is placed between the two front teeth of the patient and is single. In this case, it is better to be stretched. Because it completely messes up a person’s appearance. Sometimes there is an extra tooth at the end and it can be used instead of the original tooth by removing the previous permanent tooth. If one of the teeth in the jaw where there is an extra tooth has fallen due to a fracture, or has been pulled, we can place the extra tooth and replace the permanent tooth with orthodontics. Another issue that should be considered is whether the patient’s extra tooth has a good root or not? Sometimes the extra tooth does not have a good root. For example, it is italicized and small and is at the end. If the tooth is misaligned, it is difficult to move it [35].

### What Factors Cause Extra Teeth?

Currently, it is believed that an extra tooth may be created due to the continuous growth of the tissues that make up the teeth. Extra tooth growth may be hereditary or related to one of these two conditions: Gardner’s syndrome and cleidocranial dysplasia. It is also possible to develop extra teeth in people who do not have any of these two conditions and do not have a family history of extra teeth [36].

## Discussion

The Seals study showed that SSC is a strong and durable restoration that can be used in pulpotomy or pulpectomized deciduous teeth and teeth with developmental defects and extensive caries involving multiple tooth surfaces. Because the use of amalgam in these teeth will cause the treatment to fail. The use of SSC in caries-prone children who have decayed anterior teeth and molars causes long-term preservation of teeth. Both in terms of strength and durability, veneer is superior to double-level amalgam [37]. According to De Angelis et al.'s (2015), study, the percentage of annual failure in stress-tolerant caries in deciduous molars is 0-14% in SSC, 0-35% in amalgam restorations, 0-25-8% in glass-ionomer restorations, and 1.29% respectively. -2% in non-traumatic restorations, 0-15% in composite restorations and 0-11% in composite restorations. The main causes of failure were secondary decay, marginal defects, fractures and wear [38]. Eslami (2013), stated that dentists have been using SSCs for the restoration of deciduous and permanent posterior teeth for about 50 years. Because they have advantages such as comfort, low price, strength and reliability compared to other repairs [39].

The results of the study by Mata and his colleagues showed that the use of SSC in primary teeth leads to a longer lifespan and a reduction in retreatment. According to Konstantonis et al.'s study (2013), prefabricated metal veneers in the treatment of severely decayed primary teeth have a much higher success rate than other restorative methods [40].

**Figure:**
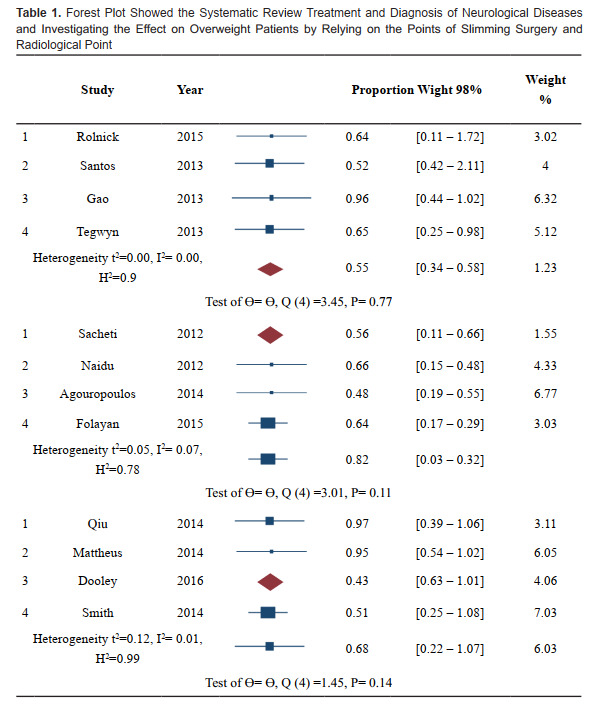


Based on Guler et al.'s study (2021), oral health professionals should use the best option in choosing the type of restoration to restore teeth in childhood. When this restoration is placed in the mouth, it should provide several goals, including strength, beauty, reduction of symptoms, etc. According to the study by Guler et al., (2021), [41] stainless steel crowns have been proposed for many years as very durable restorations with specific applications for the restoration of pulpotomy or Venkatesh et al., (2014), deciduous teeth, teeth with developmental defects, and extensive carious lesions involving multiple surfaces [42]. Since these veneers completely cover the tooth surfaces and protect against caries in the future, and because of their high durability and stability, special attention is paid to the use of SSC in children who are treated under general anesthesia. To be made Levering and Messer compared the durability and longevity of SSC and two-level amalgam restorations and found that veneers placed in children younger than 4 years and older than 4 years had approximately twice the success rate of amalgam per year for 10 years of operation by Terlemez et al., (2018) [43]. They suggested that, when given a choice between two-level amalgam restorations and SSC in a child under 4 years of age, the chance of amalgam failure is approximately twice that of these veneers. When durability is desired, this coating is the definitive choice. In another study, they compared the cost of restoring primary teeth and showed that amalgam replaced with SSC was the most expensive restoration [43]. Two other factors that are important in deciding the use of SSC are:

1- The role of parents in home care.

2- Visiting the dentist regularly to check the teeth regularly.

Malocclusion can lead to problems with biting, chewing and speech, and can also increase the risk of tooth decay and gum disease. Orthodontics plays an important role in the management of cleft lip and palate. Orthodontic treatment can help to improve dental appearance, face, and eliminate malocclusions that can harm the long-term health of teeth and periodontium. Orthodontists work closely with surgeons to restore the gum line with bone, so that the tooth roots are better supported. They also work with the prosthodontist for dental implants and braces. In some cases, they may even offer Nasal Alveolar Molding (NAM) to mold the lips, palate, and nose prior to cleave lip repair. If there is a crossbite, a palate expander may be needed before braces.

In a crossbite, the upper teeth are narrower than the lower teeth and may be inside or behind the lower teeth. Laganà et al. (2017), during a joint project by a group of doctors affiliated with various international associations such as the American Board of internal medicine [44], the American college of physicians and the European federation of internal medicine, a medical charter on professionalism was designed and published. In this regard, the aforementioned doctors introduced three fundamental principles that characterize professionalism, which are:

* Prioritizing the patient and his well-being.

* Giving the patient the right to decide and social justice.

* They also presented 10 series of responsibilities that today's doctors should be committed to, which include:

* Have professional competence and be able to maintain it.

* Be honest with patients.

* Be confidential about the patient.

* Avoid establishing inappropriate relationships with patients.

* Expand their scientific knowledge.

* Promote fair distribution of resources.

* Through the correct management of the conflict of interest, gain the confidence of patients [45].

## Conclusion

Closing a cleft lip with surgery is easier than repairing a cleft palate. Cleft lip and palate surgery is usually performed within three to four months after birth, and generally the scar left by it disappears as the child grows, but cleft palate surgery is postponed until the child is one to two years old. Because at this time the upper jaw has reached normal growth. If the damage is extensive, surgery is delayed until 5-7 years of age to prevent structural problems. In some cases, surgery is not possible or cannot completely close the gap. In some cases, a device similar to an artificial tooth called an obturator is made to cover the gap and allow the child to eat normally. Depending on the severity of the cleft palate, multiple surgeries may be needed over a long period of time. A plastic surgeon or maxillofacial surgeon performs reconstructive surgery on the face. Despite the advances made in the field of cleft lip surgery techniques and corrective devices, very good results can be expected for children with cleft lip and palate, so that usually, as these children grow up, a small trace of the cleft remains. Orthodontics is one of the most effective methods for treating extra teeth. In some cases, extra teeth can benefit the patient. For example, if a person has lost one of his main teeth and has an extra tooth in the back of the mouth, the extra tooth can be used as a replacement for the main tooth. Children with cleft lip and palate often face oral health problems. For example, they may be born with a missing tooth or an extra tooth. These issues can lead to various dental abnormalities such as discontinuity of the alveolar process, loss and malformation of teeth and malocclusion (misalignment of teeth).

## Conflict of Interest

None.
